# Prevalence of gastrointestinal parasitism in small ruminants in western zone of Punjab, India

**DOI:** 10.14202/vetworld.2017.61-66

**Published:** 2017-01-17

**Authors:** E. Singh, P. Kaur, L. D. Singla, M. S. Bal

**Affiliations:** 1 Department of Veterinary Parasitology, Guru Angad Dev Veterinary and Animal Sciences University, Ludhiana - 141 004, Punjab, India; 2 Animal Disease Research Centre, Guru Angad Dev Veterinary and Animal Sciences University, Ludhiana - 141 004, Punjab, India

**Keywords:** gastrointestinal parasitism, goat, prevalence, Punjab, sheep, western zone

## Abstract

**Aim:**

The aim of this study was to explore the prevalence of gastrointestinal parasitism in small ruminants in relation to various risk factors in the western zone of Punjab.

**Materials and Methods:**

During the study, 603 fecal samples (391 of sheep and 212 of goats) were examined qualitatively by floatation and sedimentation techniques, and quantitatively by McMaster technique.

**Results:**

Out of the 603 fecal (391 sheep and 212 goats) samples examined, 501 were found positive for endoparasitic infection with an overall prevalence of 83.08%, consisting of 85.16% and 79.24% in sheep and goats, respectively. Egg per gram in sheep was apparently more 1441.88±77.72 than goats 1168.57±78.31. The associated risk factors with the prevalence of gastrointestinal tract (GIT) parasites showed that females (85.97%) were significantly more susceptible than males (69.23%). Age wise the adults (>6 months) were significantly more prone to parasitic infection as compared to young ones (<6 months). Seasonal variation was recorded throughout the year and was significantly highest during monsoon (90.10%), followed by winter (83.84%) and summer (78.35%).

**Conclusion:**

The study revealed an overall prevalence of 83.08% of GIT parasitic infections in small ruminants constituting 85.16% in sheep and 79.24% in goats in the western zone of Punjab. The most relevant risk factors for the prevalence of gastrointestinal parasitism in ruminants were sex, age, and season.

## Introduction

Small ruminants hold an important niche for sustainable agriculture in developing countries and support a variety of socioeconomic functions worldwide. India has an estimated sheep and goat population of 65 million and 135.17 million, respectively, whereas Punjab has 0.15 million sheep and 0.32 million goats as per 19^th^ livestock census [1]. Gastrointestinal tract (GIT) parasitism in sheep and goats is of paramount importance because small ruminants’ rearing has been a major source of income especially to the marginal farmers of the country [2]. These parasites cause both acute infections with a rapid onset and high mortality levels and chronic infections, which are commonly subclinical and may lead to insidious and important economic losses [3] via reduction of live weight gain, reduced wool and milk production, and poor reproductive performance [4]. This problem is severe in tropical countries due to highly favorable environmental conditions for helminth transmission [5].

Studies dealing with the distribution and parasite control measures adopted by small landless marginal farmers in the Punjab state are very limited or absent, especially in the western zone.

Present study aimed to identify the prevalence and risk factors associated with ovine and caprine GIT parasites, which is vital for future holistic prevention and control strategies in the area.

## Materials and Methods

### Ethical approval

This study was based on the fecal sample collection only, hence the ethical approval was not required. The fecal samples were directly collected from the animals without any harm or freshly voided samples with the prior consent of the owners.

### Study area

Punjab state extends from the latitudes 29°30’ N to 32°32’ N and longitudes 73°55’ E to 76°50’ E in the northwest region of India. It covers a geographical area of 50,362 km^2^, which is 1.54% of country’s total area and lies between altitudes 180 m and 300 m above mean sea level. Average rainfall in Punjab is 565.9 mm and ranges from about 915 mm in north to 102 mm in south. The state has been classified into five agro-climatic zones on the basis of homogeneity, rainfall pattern and distribution, soil texture, cropping patterns, etc. Western zone constitute of six districts, *viz*., Barnala, Bathinda, Mansa, Moga, Muktsar, and Sangrur having average annual rainfall of <400 mm, which is considered to be the hottest and drier zone of Punjab.

### Sample collection and fecal analysis

A total of 603 (391 of sheep and 212 of goats) fecal samples were randomly collected directly from the rectum of animals or freshly voided during the period of March 2015 to May 2016 in each season uniformly from six districts of western zone. Samples were labeled accordingly and stored in ice chilled container to slow down the process of nematode eggs development during transportation. The samples were grossly examined for color, consistency, odor and for the presence of adult worms or developmental stages, if any. The fecal samples were processed and screened qualitatively using sedimentation and floatation methods for evaluating the incidence of infections. The quantitative examination or egg per gram (EPG) estimation was done as per McMaster technique [6]. A questionnaire was prepared for the prevalence in terms of various risk factors, *viz*., species, age, sex and season, type of management, and treatment given based on the history taken at the time of sampling.

### Statistical analysis

Data analysis was performed using Statistical Analysis System (SAS for Windows, Version 9.4, USA). Association between the prevalence of GIT helminth infections and various factors was carried out by Chi-square test (*χ*^2^ – test).

## Results

In this study, out of 603 fecal samples examined, 501 were found positive with an overall prevalence of 83.08% for GIT parasitic infections (Table-1) indicating district wise significantly (p<0.05) highest prevalence in Sangrur (88.78%) and lowest in Bathinda (68.08%). The ovine (85.16%) were apparently more susceptible to the GIT parasitic infections than caprine (79.24%) (Table-2). The district wise prevalence of GIT parasites in sheep and goat is given in Table-3. Similarly, to an overall prevalence of the GIT parasites in small ruminants, individually prevalence in both the species was highest in Sangrur district. The parasitic load in terms of mean EPG in sheep was apparently more 1441.88±77.72 than goats 1168.571±78.31. The parasite-wise distribution among the two species showed that only strongyle was significantly high in sheep (39.63%) than goats (19.04%). Sex wise an overall copro-prevalence of GIT parasites in both the species showed that females (85.97%) were significantly (p<0.01) more susceptible than males (69.23%) (Table-4). In this study, the animals were divided into two age groups, *viz*., young (<6 months) and adults (>6 months). Age wise an overall prevalence between young and adult group showed that adults (>6 months) were significantly more prone to parasitic infection with the prevalence of 85.97%. In sheep, the results showed that an overall copro-prevalence of different age group was found to be significantly (p<0.01) higher in adults (89.73%) as compared to young animals (54.00%) (Table-5). However, in goats, a nonsignificant difference was observed between young ones (71.42%) and adults (80.79%). The data collected in different months were partitioned according to season, *viz*., Monsoon (July to October), winter (November to February), and summer: (March to June) (Table-6). Season wise copro-prevalence of GIT parasitic infections in both the species was significantly (p<0.01) highest in monsoon (90.10%), followed by winter (83.84%) and lowest in summer (78.35%). The quantitative parasitic load based on the mean values and standard error of EPG of helminth infection was highest in monsoon followed by winter and then summer (Table-7). The degree (severity) of helminth parasitic infection was determined from the EPG count. Out of 603 samples, 36.31% were infected lightly (EPG range 100-1000) and 3.64% were found highly positive with mean EPG range >4000. The animals with fecal egg count in the range of 1000-2000 were 23.21%, between 2000 and 3000 were 8.78% and only few proportions of animals had fecal egg count of 3000-4000 (1.65%) (Table-8).

**Table-1:** District wise prevalence of GIT parasites in small ruminants of western zone of Punjab.

District	Number of samples examined	Positive samples (%)			Total positive samples (%)

		Single parasitic infection	Dual parasitic infection	Mixed parasitic infection^#^	
Barnala	117	46 (39.31)	43 (36.75)	8 (7.47)	97 (82.90)
Bathinda	47	27 (57.44)	5 (10.63)	-	32 (68.08)
Mansa	129	42 (32.55)	56 (43.41)	6 (4.65)	104 (80.62)
Moga	106	51 (48.11)	35 (33.01)	6 (5.66)	92 (86.79)
Muktsar	97	24 (24.74)	48 (49.48)	9 (9.27)	81 (83.50)
Sangrur	107	31 (28.97)	49 (45.79)	15 (14.01)	95 (88.78)
Total	603	221 (36.65)	236 (39.13)	44 (7.29)	501 (83.08)
χ^2^ value					11.607*

* indicates values varying significantly at p<0.05.

# Mixed parasitic infections contains more than two parasites *viz*. Strongyle+*Strongyloides*+Coccidia, Strongyle+Coccidia+Amphistome, Strongyle+Coccidia+*Strongyloides*+Amphistome, Strongyle+Coccidia+*Strongyloides*+*Moniezia*+*Trichuris*, etc., GIT=Gastrointestinal tract

**Table-2:** Comparative species wise copro-prevalence and quantitative load of GIT parasites of small ruminants in western zone of Punjab.

Species	Total samples examined	Total positive (%)	Single parasitic infection (%)		Dual parasitic infection (%)					Mixed infection (more than two species)^#^ (%)	Mean EPG±standard error
	
			Str.	Coc.	Str. +Coc.	Str. + Mon.	Str. + Strg.	Str. + Tri.	Str. + Amp.		
Sheep	391	333 (85.16)	132 (39.63)	29 (8.70)	134 (40.24)	6 (1.80)	6 (1.80)	4 (1.20)	1 (0.30)	21 (6.30)	1441.88±77.72
Goat	212	168 (79.24)	32 (19.04)	28 (16.66)	75 (44.64)	3 (1.78)		3 (1.78)	4 (2.38)	23 (13.69)	1168.57±78.31
χ^2^ value	3.429	21.504*	7.014	0.890		3.064	0.277	4.893**	7.600*	

* indicates values varying significantly at p<0.01,

** indicates values varying significantly at p<0.05.

# Mixed infections (Strongyle+*Strongyloides*+Coccidia=12; Strongyle+*Moniezia*+Coccidia=18; Strongyle+*Strongyloides*+*Moniezia*=1; Strongyle+Coccidia+Amphistome=3; Strongyle+Coccidia+*Trichuris*=5; Strongyle+*Moniezia*+Amphistome=1; Strongyle+Coccidia+*Strongyloides*+Amphistome=2; Strongyle+*Moniezia*+Coccidia+*Strongyloides*=1; Strongyle+Coccidia+*Strongyloides*+*Moniezia*+*Trichuris*=1). Str=Strongyle, Coc=Coccidia, Tri=*Trichuris*, Amp.=Amphistomes, Mon.=*Moniezia*, Strg=*Strongyloides*, GIT=Gastrointestinal tract

**Table-3:** District wise copro-prevalence of gastrointestinal parasites in sheep and goats in western zone of Punjab.

Districts	Sheep		Goat	
	
	Total samples examined	Total positive (%)	Total samples examined	Total positive (%)
Barnala	96	80 (83.33)	21	17 (80.95)
Bathinda	19	16 (84.21)	28	16 (57.14)
Mansa	101	82 (81.18)	28	22 (78.57)
Moga	65	58 (89.23)	41	34 (82.92)
Muktsar	66	56 (84.84)	31	25 (80.64)
Sangrur	44	41 (93.18)	63	54 (85.71)
Total	391	333 (85.16)	212	168 (79.24)
χ^2^	4.627		10.339	

**Table-4:** Sex based copro-prevalence of GIT parasites in small ruminants.

Species	Males		Females		Total		χ^2^
		
	Number of samples examined	Positive samples (%)	Number of samples examined	Number of samples positive (%)	Examined	Positive	
Sheep	58	42 (72.41)	333	291 (87.38)	391	333 (85.16)	8.767*
Goat	46	30 (65.21)	166	138 (83.13)	212	168 (79.24)	7.029*
Total	104	72 (69.23)	499	429 (85.97)	603	501 (83.08)	
χ^2^	17.163*						

* indicates values varying significantly at p<0.01. GIT=Gastrointestinal tract

**Table-5:** Age wise copro-prevalence of GIT parasites in sheep in western zone of Punjab.

Species	<6 months		>6 months		Total		χ^2^
		
	No of samples examined	Positive samples (%)	No of samples examined	No of samples positive (%)	Examined	Positive	
Sheep	50	27 (54.00)	341	306 (89.73)	391	333 (85.16)	44.080*
Goat	35	25 (71.42)	177	143 (80.79)	212	168 (79.24)	1.557
Total	85	52 (61.17)	518	429 (85.97)	603	501 (83.08)	
χ^2^	21.191*						

* indicates values varying significantly at p<0.01. GIT=Gastrointestinal tract

**Table-6:** Season wise copro-prevalence of GIT parasitic infection in small ruminants.

Season	Sheep			Goat			Both		
		
	Number of samples examined	Number found positive	Infection (%)	Number of samples examined	Number found positive	Infection (%)	Number of samples examined	Number found positive	Infection (%)
Winter	79	69	87.34	51	40	78.43	130	109	83.84
Summer	203	165	81.28	88	63	71.59	291	228	78.35
Monsoon	109	99	90.82	73	65	89.04	182	164	90.10
χ^2^		5.485			7.414*			11.086**	

* indicates values varying significantly at p<0.05.

** indicates values varying significantly at p<0.01. GIT=Gastrointestinal tract

**Table-7:** Season wise mean fecal egg count and standard error of helminths in small ruminants of western zone of Punjab.

Species	Season		

	Monsoon	Winter	Summer
Sheep	1640.99±122.01	1298.21±154.92	1069.31±99.59
Goat	1507.81±131.74	908.88±105.68	845.16±121.00

**Table-8:** Quantification of nematode eggs in small ruminants.

Species	Number of samples examined	Number of positive samples (%) within EPG range				

		100-1000	1000-2000	2000-3000	3000-4000	>4000
Sheep	391	147 (37.59)	95 (24.29)	32 (8.18)	10 (2.55)	20 (5.11)
Goat	212	72 (33.96)	45 (21.22)	21 (9.90)	-	2 (0.94)
Total	603	219 (36.31)	140 (23.21)	53 (8.78)	10 (1.65)	22 (3.64)

EPG=Egg per gram

## Discussion

There was slight variation in prevalence of GIT parasitic infection among five districts except Bathinda. The lowest prevalence in Bathinda district may be attributed to the fact that most of the animals examined were kept in confinement and managed on intensive system management. They were having restricted access to outer infection sources and were dewormed regularly as suggested by the veterinarian. In contrast in district Sangrur, the highest prevalence may be due to the fact that the field flocks of sheep and goats encountered during the study were mainly from the nomadic farmers that kept on changing the pastures, thus had an access to abundant of various parasitic egg/ova prevailing in these areas and rarely they preferred deworming their animals. District wise, the single parasitic infection was higher in Bathinda (57.44%), while the dual infection was high in Muktsar (49.48%) and multiple infections having more than three parasites were high in Sangrur district (14.01%). The high incidence of single infection in Bathinda district may be due to the fact that encountered animals were reared on the intensive grazing system.

The results of the species-wise prevalence (Tables-2 and 4) revealed that the sheep was more susceptible to helminth infection than goats. Similar observations were reported in different states of India [5-9]. Higher prevalence of GIT parasitic infections in sheep as compared to goats was probably due to their grazing behavior. Sheep grazes very close to the ground so risk of ingestion of parasitic ova is comparatively more than the goats, as they are browsers [10]. In contrast to the present findings, higher rates of infection throughout the year in goats were reported [11,12]. This variation in prevalence depends on the difference in agro-climatic condition and availability of susceptible host [5].

During the present study, it was found that overall prevalence of parasitic infection was significantly higher in females than their counterpart males. Among sheep, a significantly (p<0.01) higher prevalence was recorded in females (87.38%) as compared to their male counterparts (72.41%). Similarly to sheep, the infection in goats was found to be significantly (p<0.01) higher in females (83.13%) than males (65.21%). The influence of sex on the susceptibility of animals to infections could be attributed to genetic predisposition and differential susceptibility owing to hormonal control. The physiological peculiarities of the female animals, which usually constitute stress factors thus reducing their immunity to infections, and for being lactating mothers, females happen to be weak and malnourished, as a result of which they are more susceptible to the infections besides some other reasons [13,14].

The current study revealed that the adults were significantly more prone to parasitic infection with the prevalence of 85.97% than the young ones. It could be explained that higher nematode prevalence in adults might be due to grazing on larger area of pastures being contaminated with various flocks and different stress conditions such as climate, long daily traveling, and gestation [15]. The young animals are less susceptible to parasitic infections due to less exposure for grazing as they mainly depend upon milk feeding. Our findings were in concordance with Yadav *et al*. [16], Emiru *et al*. [17] who recorded a higher prevalence of infection in adults than young ones.

Out of the three seasons, the highest prevalence of parasitic infection was recorded in monsoon followed by winter and then summer. The findings are in consistent with the various published reports [8,18,19]. The reason for higher prevalence in monsoon could be attributed from the fact that favorable climatic conditions, *viz*., humidity and temperature, supports parasitic growth, and development led to increased availability of infective larvae in this season. It is well documented that GIT parasitism in grazing animals is directly related to the availability of larvae on pasture and seasonal pasture contamination [19]. Climatic factors also influence in larval dispersion on the herbage which increases the chance of contact between host and larvae. Higher rate of infection in monsoon may also be attributed to suitable molarities of salt present in soil which is an important factor for ecdysis [20]. Such climatic conditions also help in bacterial multiplication which provides nutrition to free-living larvae. Moreover, high prevalence in adults and in summer season can coincide with the fact that lambing and kidding in the study area normally occurs in the month of February (winter season) and in the month of October (monsoon season). Periparturient rise of eggs counts may be responsible for overall rise of infection rate during these seasons in summer season. In contrast to current findings, the highest prevalence of GIT parasites during monsoon followed by summer and winter was reported [15]. Hutchinson *et al*. [21] reported that cold stimulus is responsible for arrested development of larvae. During winter, animals are also partially stall-fed that reduces chance of infection. Period of grazing is also reduced during winter as well as pre-parasitic stages also undergo hypobiosis which also contributes to low infection during this period. The majority of ewes are pregnant during this period. Hormonal impact results in low fecal egg output and contributes to low availability of infection in pastures.

About the levels of EPG to be considered as pathogenic, there is a wide variation in the opinion of researchers and no firm limit has been fixed for lower and upper EPG range. In an experimental study [22] categorized resistant goats with EPG range 250-1800 and susceptible with EPG range of 5400-14,900, while Palamapalle *et al*. [23] reported 6023 EPG (3000-105,000) in subclinical nematode infection. This study revealed that prevalence of nematode infection was not associated with clinical form though increase in the EPG count is positively correlated with worm burden [18]. Anthelminthic resistance also influence prevalence and egg counts [24].

## Conclusions

Keeping in view the present findings, it can be concluded that the high prevalence rate of gastrointestinal parasitism needs to be monitored periodically among the small ruminants not only in western zone but in the other zones of the state as well. Further, effective and well-planned control measures to check the parasitic population should be implicated by conducting extension programs to educate the farmers regarding the proper use of anthelmintics.

## Authors’ Contributions

PK, LDS, and MSB helped in the collection of samples, planning, designing and supervising the research work. ES collected the samples and conducted the experiment. All authors read and approved the final manuscript.

## References

[1] DAHD and F. (2012) 19 Livestock Census-2012 All India Report.

[2] Pathak A.K, Pal S (2008). Seasonal prevalence of gastrointestinal parasites in goats from Durg district of Chhattisgarh. Vet. World.

[3] Singla L.D (1995). A note on sub-clinical gastro-intestinal parasitism in sheep and goats in Ludhiana and Faridkot districts of Punjab. Indian Vet. Med. J.

[4] Sutherland I, Scott I (2010). Gastrointestinal Nematodes of Sheep and Cattle: Biology and Control.

[5] Singh V, Varshney P, Dash S.K, Lal H.P (2013). Prevalence of gastrointestinal parasites in sheep and goats in and around Mathura, India. Vet. World.

[6] Gupta S.K, Singla L.D, Gupta R.P, Garg S.R, Nehra V, Lather D (2012). Diagnostic trends in parasitic diseases of animals. Veterinary Diagnostics: Current Trends.

[7] Varadharajan A, Vijayalakshmi R (2015). Prevalence and seasonal occurrence of gastrointestinal parasites in small ruminants of coastal areas of Tamil Nadu. Int. J. Sci. Res. Publ.

[8] Velusamy R, Rani N, Ponnudurai G, Anbarasi P (2015). Prevalence of intestinal and haemoprotozoan parasites of small ruminants in Tamil Nadu, India. Vet. World.

[9] Singh R (2015). Epidemiology of gastrointestinal parasites of sheep and goats in central plain zone of Punjab.

[10] Lathamani V.S, Ramesh P.T, Siddalingamurthy H.K (2016). Studies on the prevalence of helminth infestation in small ruminants and the anthelmintic effectiveness in Tumkur District of Karnataka. Int. J. Innov. Res. Sci. Eng. Technol.

[11] Pandey V.S, Ndao M, Kumar V (1994). Seasonal prevalence of gastrointestinal nematodes in communal land goats from high yield of Zimbabwe. Vet. Parasitol.

[12] Saha S.B, Pramanik S, Mukherjee G.S (1996). Prevalence of gastrointestinal nematodes of goats in West Bengal. Indian J. Anim. Sci.

[13] Blood D.C, Radostitis O.M (2000). Veterinary Medicine.

[14] Mir M.R, Chishti M.Z, Majidah R, Dar S.A, Katoch R, Khajuria J.K, Mehraj M, Dar M.A, Rasool R (2013). Incidence of gastrointestinal nematodosis in sheep of Jammu. Trends Parasitol. Res.

[15] Radostits O.M, Blood D.C, Gay C.C (1994). Veterinary Medicine.

[16] Yadav A, Khajuria J.K, Raina A.K (2006). Seasonal prevalence of gastrointestinal parasites in sheep and goats of Jammu. J. Vet. Parasitol.

[17] Emiru B, Amede Y, Tigre W, Feyera T, Deressa B (2013). Epidemiology of gastrointestinal parasites of small ruminants in Gechi District, Southwest Ethiopia. Adv. Biol. Res.

[18] Dhara K.C, Bandopadhyay P.K, Goswami A (2011). Influence of gastro-intestinal parasites on the productive and reproductive performances of Black Bengal goat under field conditions. Int. J. Sci. Nat.

[19] Gaherwal S, Prakash M.M, Dudwe J (2016). Prevalence and incidence of nematodes in goats at five different villages of Barwani district, Mathya Pradesh. Int. J. Adv. Res.

[20] Soulsby E.J.L (1966). Biology of Parasites.

[21] Hutchinson G.W, Lee E.H, Fernando M.A (1972). Effects on variation in temperature on infective larvae and their relationship to inhibited development of *Obeliscoides cuniculi* in rabbit. Parasitology.

[22] Chartier C, Hoste H (1998). Repeated infection with *Haemonchous contortus* and *Trichostrongylus* columbiformis in dairy goats: Comparison of resistant and susceptible animals. Parasitol. Res.

[23] Palamapalle H, Deshpande P.D, Narladkar B (2003). Gastro-intestinal nematodiasis in bovines of Marathwada region: Faecal culture and egg count studies. J. Vet. Parasitol.

[24] Singh R, Bal M.S, Singla L.D, Kaur P (2016). Detection of anthelmintic resistance in sheep and goat against fenbendazole by faecal egg count reduction test. J. Parasit. Dis.

